# 

**Published:** 1896-06

**Authors:** 


### Communicated.

### NATIONAL ASSOCIATION OF DENTAL FACULTIES.

The annual meeting of the National Association of Dental Faculties will be held at Saratoga Springs, on Saturday, August 1st, at 10 o'clock, a. m. The Executive Committee will meet on Friday July 31st, at 10 o'clock a. m. Those having matters to bring before this Committee will do well to bear this meeting in mind. The work of this Committee is preparatory to that of the general meeting. All communications requiring the attention of the Executive Committee should be sent to the Chairman.

DR. J, TAFT,
Chairman Executive Committee,
Berkshire Building, Cincinnati.

DR. LOUIS OTTOFY, Secretary,
Masonic Temple, Chicago.

### ILLINOIS STATE DENTAL SOCIETY.

The thirty-second annual meeting of the Illinois State Dental society was held at Springfield, May 12 to 15, 1896, A good program was carried out, and a large attendance was present. The following officers were elected:

President, C. R. Taylor; Vice-President, E. B. David, Aledo; Sec'y, Louis Ottofy, Chicago; Treas., E. D. Swain. Chicago; Librarian, J. R. Rayburn, Fairbury. The next annual meeting will be held at Peoria, beginning on the second Tuesday in May, 1897.

LOUIS OTTOFY, Secretary,
Masonic Temple, Chicago.

### THE CHICAGO DENTAL SOCIETY.

The following is the list of Officers of the Chicago Dental Society for 1896-7, elected at the annual meeting, held in Columbus Memorial Building, Tuesday evening, April 7th, 1896:

President, Louis Ottofy; First Vice-President, J. E. Hinkins; Second Vice-President, H. A. Costner; Recording Secretary, A. H. Peck; Corresponding Secretary, Geo. B. Perry; Treasurer, E. D. Swain; Librarian, H. A. Gunther; Member Board of Directors, G. H. Cushing; Board of Censors, C. T. Carpenter, B. D. Wikoff, G. W. Schwartz.

### THE AMERICAN DENTAL SOCIETY OF EUROPE.

The American Dental Society of Europe will hold its 21st meeting at Dresden, Germany, Aug. 3d, 4th, and 5th, 1896. All members of the profession who plan to be in Europe at that time are cordially invited to attend.

Further information can be obtained of the President,
DR. JOHN H. SPAULDING, Paris, or of
WILLIAM A. SPRING,
26 Christian St., Dresden.

### AMERICAN DENTAL ASSOCIATION.

The annual meetings of the American Dental Association will be held at Saratoga Springs, N. Y., July 31–August 7, 1896. The regular meetings of the American will begin Tuesday, August 4th.

The Grand Union Hotel has been selected as the headquarters and the meetings will be held there. Ample space for committee rooms and exhibits has been secured. The ball-room and club-house, connecting with ball-room, have also been placed at our disposal.

The railroad arrangements have not yet been completed but we expect to secure the usual fare-and-a-one-third rate. Tickets good three days before date of meeting and three days after date of closing. Dentists should pay full fare in going and take a receipt therefor, as it will be impossible to secure the one-third rate in returning unless you hold such receipt.

J. N. CROUSE,
Chairman Executive Committee.

---

### THE NATIONAL ASSOCIATION OF DENTAL EXAMINERS.

The twelfth annual session will be held at Saratoga Springs, N. Y., commencing 10 o'clock, a. m., Monday, August 3d, and continue in session during the proceedings of the American Dental Association.

It is earnestly requested that all State and Territorial Boards of Dental Examiners will send delegates.

CHAS. A. MEEKER, Sec'y. and Treas.
29 Fulton Street,
Newark, N. J.

### To Readers and Correspondents.

Communications intended for publication, and exchange journals and papers, should be addressed to the Editor of The American Journal of Dental Science, No. 845 N. Eutaw St. Baltimore, Md. All letters relating to the business of the Journal, or containing cash remittances, to be directed to Snowden & Cowman Mfg. Co., 9 W. Fayette Street, Baltimore, Md. Articles for publication should be received by the Editor at least two weeks previous to the first of the month on which the number in which it is designed for them to appear is issued.

☞ The American Journal of Dental Science will contain fifty pages of reading matter in each number, making a volume of about Six Hundred pages,

EXCLUSIVE OF ADVERTISEMENTS.

